# Risk Prediction Modeling of Sequencing Data Using a Forward Random Field Method

**DOI:** 10.1038/srep21120

**Published:** 2016-02-19

**Authors:** Yalu Wen, Zihuai He, Ming Li, Qing Lu

**Affiliations:** 1 Department of Statistics, University of Auckland, Auckland 1010, New Zealand; 2 Department of Biostatistics, University of Michigan, Ann Arbor, Michigan 48109, U.S.A; 3 Department of Epidemiology and Biostatistics, Indiana University at Bloomington, Bloomington, IN 47405, U.S.A; 4 Department of Epidemiology and Biostatistics, Michigan State University, East Lansing, MI 48824, U.S.A.

## Abstract

With the advance in high-throughput sequencing technology, it is feasible to investigate the role of common and rare variants in disease risk prediction. While the new technology holds great promise to improve disease prediction, the massive amount of data and low frequency of rare variants pose great analytical challenges on risk prediction modeling. In this paper, we develop a forward random field method (FRF) for risk prediction modeling using sequencing data. In FRF, subjects’ phenotypes are treated as stochastic realizations of a random field on a genetic space formed by subjects’ genotypes, and an individual’s phenotype can be predicted by adjacent subjects with similar genotypes. The FRF method allows for multiple similarity measures and candidate genes in the model, and adaptively chooses the optimal similarity measure and disease-associated genes to reflect the underlying disease model. It also avoids the specification of the threshold of rare variants and allows for different directions and magnitudes of genetic effects. Through simulations, we demonstrate the FRF method attains higher or comparable accuracy over commonly used support vector machine based methods under various disease models. We further illustrate the FRF method with an application to the sequencing data obtained from the Dallas Heart Study.

Benefiting from the new technologies, great progress has been made through genome-wide association studies (GWAS) in identifying common variants associated with complex diseases^1^. With the emerging genetic findings, studies have been conducted to assess the role of disease-associated genetic markers in early disease prediction. However, risk prediction models formed to date have low utility for clinical use^2^,^3^,^4^,^5^. The poor performance could be due to the use of only a limited number of common variants, with significant but often small marginal effects^2^. Because the majority of genetic markers, especially rare variants, have not yet been fully studied in recent risk prediction studies, the natural next step will be to study the role of additional genetic variants, including rare variants, in disease prediction. Recent studies suggest that rare variants can play an important role in the genetic etiology of complex diseases. For example, it has been shown that rare variants were associated with autisms, atherosclerosis and mental retardation^6^,^7^,^8^,^9^. Evolutionary theory also suggests that recent rare variants could be more deleterious than common variants because they are under less negative selective pressures.

Although promising, incorporating rare variants into risk prediction models remains great challenges. The conventional statistical methods are subject to poor performance because of the large number and the low frequency of rare variants. Recently, gene-based approaches have been developed for genetic association analysis of sequencing data. It has been argued by Neale and Sham that gene-based approaches have several advantages over single-locus approaches^10^,^11^, as it is well known that gene is a functional unit of the human genome^10^. Furthermore, by jointly evaluating all single nucleotide variants (SNVs) within a putative gene, gene-based approaches are capable of aggregating association signals from multiple SNVs, reducing the number of tests, and incorporating rare variants into the analysis^12^,^13^. The same idea can be applied to risk prediction analysis, where the cumulative effect of SNVs within a gene can be evaluated. In addition, it is important to consider multiple genes and to select the disease-associated genes while building a risk prediction model. It has been shown by Byrnes *et al.* that in the absence of good annotation, variable selection algorithms could substantially improve the performance of the model^14^.

Many gene-based approaches can be extended for risk prediction modeling. Among those, random field based methods have been shown to have nice properties and have been popularly used in spatial analysis and imaging analysis for prediction purposes^15^. Nevertheless, it has not been used for high dimensional genetic risk prediction. In this study, we develop a forward random field (FRF) method for risk prediction modeling of sequencing data. The FRF method adopted a forward selection algorithm to search for the disease-associated genes, and estimated the effects of the selected genes through solving generalized estimating equations. The proposed method was compared to support vector machine (SVM) based methods, which aimed at building risk prediction models with consideration of both common and rare variants^4^. We further illustrated the proposed method through an application to a sequencing dataset from the Dallas Heart Study (DHS).

## Method

The FRF method is motivated by the general idea in spatial statistic that the adjacent points in the space share similar outcomes^16^,^17^,^18^. In the FRF method, we assume that the adjacent individuals with similar genotypes have more similar phenotypes than distant individuals, where the distance between two individuals is gauged based on a pre-defined distance function. Under this assumption, the phenotype of an individual is modelled as a linear function of the other subjects’ phenotypes weighted by the genetic similarities of their genes. When multiple genes with different magnitudes and directions of effects are considered, the weights will be determined by the genetic similarities of each gene.

Consider a case-control study of *N* individuals and a total of *L* SNVs located on *K* genes, where each gene has 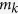 SNVs, i.e., 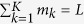. Let *Y*_*i*_ be the response measurement for the *i*^th^ individual with *Y*_*i*_ = 0 for the unaffected status and *Y*_*i*_ = 1 for the affected status. Let 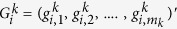 be the genotypes of *m*_*k*_ SNVs located on the *k*^*th*^ gene, 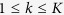, where 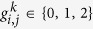. We further denote *M* covariates for individual *i* as 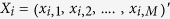.

If the *k*^*th*^ gene is associated with the phenotype, the genetic similarity measured by the *m*_*k*_ SNVs within the *k*^*th*^ gene between two subjects would lead to their phenotypic similarity. Therefore, the phenotype of the *i*^*th*^ individual could be predicted by those individuals carrying similar genetic variants within the *k*^*th*^ gene. Using the random field framework, we model the phenotype of the *i*^*th*^ individual as a linear function of the other individual’s phenotypes,


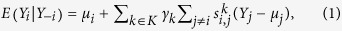


where *Y*_−*i*_ denotes the phenotypic values for all individuals excluding the *i*^th^ subject; 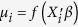 denotes for the mean function as defined in a generalized linear model, where 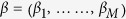 is a vector of regression coefficients for the *M* covariates. For the binary phenotype, we use the logit link
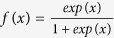
. *γ*_*k*_ is a fixed effect that measures the predictive effect of the *k*^*th*^ gene on the phenotype. 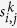 measures the genetic similarity of the *k*^*th*^ gene between individual *i* and individual *j*. It is defined as 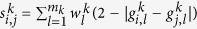, where 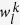 is the weight for marker *l* on the *k*^*th*^ gene defined based on minor allele frequency and 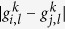 is the absolute distance between genotypes of individual *i* and individual *j* at marker *l* (Table 1).

We further denote 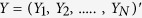, 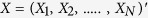, and 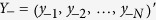, the equation (1) can be re-written in a matrix format,


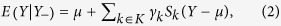


where *S*_*k*_ is an *N* × *N* the similarity matrix with zeros on the diagonal and 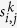 on the *i*^th^ row and the *j*^th^ column. The parameters *γ*_*k*_ in equation 2 can be estimated by solving the following unbiased estimating equations:


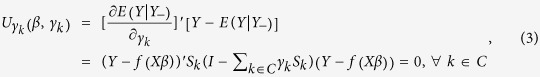


where the regression coefficients (*β*) are usually unknown, and can be estimated from the generalized linear model under the assumption of 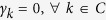. Given the estimates of *β* and *γ*_*k*_, the predicted value of a new subject’s phenotype is


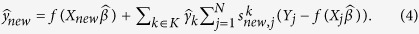


The accuracy of the risk prediction model can then be estimated using the area under the receiver operating characteristic curve (AUC),


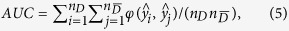


where *n*_*D*_ and 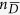 are the number of cases and controls, respectively. The kernel function *φ* is defined as, 
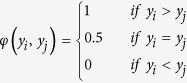
.

In genetic studies of complex diseases, the underlying genetic causes are unknown in advance. Therefore, it is quite likely a significant proportion of genes included in the study are not disease-related. Even for the disease-related genes, the underlying disease model is not clear. For instance, we have limited knowledge of whether the common variants or rare variants in genes play a more important role in disease development. Therefore, it is difficult for us to pre-specify a weight function to reflect the importance of variants. To account for the unknown disease model and to reduce the effect of noise genes, we propose a computationally feasible method, the forward selection algorithm, to simultaneously select the disease-related genes and the corresponding weight functions for the risk prediction model. The details of the algorithm are illustrated in Fig. S1. The algorithm starts from a null model and gradually adds each gene with the optimal weight function into the model. In step one, we evaluate each gene and all possible weight functions, and fit each model based on equation 4. For each model, we evaluate the classification accuracy (i.e., *AUC*) of the models using equation 5. The gene and its corresponding weight that attain the maximum *AUC* are selected into the model. In step two, based on the model selected from step one, we further evaluate the remaining gene and all possible weight functions, and select the second gene and the corresponding weight function with the maximum *AUC* into the model. The process continues until a parsimonious model with optimum number of genes, which is determined through a *K*-fold cross-validation procedure, is obtained.

## Results

### Simulations

Simulation studies were conducted to evaluate the performance of the proposed method and compared it with two existing methods, a SVM method and a modified SVM method (MSVM)^4^ for sequencing data. The MSVM method first conducts an association test for each common variant using the Fisher’s exact test, and includes those common variants with p-value less than a pre-specified threshold (e.g., p-value = 0.001) for further analysis. Using SVM, the MSVM builds a risk prediction model with the pre-selected common variants and rare variants (MAF < 0.05) collapsed based on the CMC method^19^.

The *AUC*s of three methods were compared based on 1000 replicates under various disease models, causal/non-causal SNV ratios, and the number of non-causal genes. In all the simulations described below, the genotype data were drawn from the 1000 genome project^20^. In particular, we randomly selected a 2 Mb region from the genome (i.e. chr1: 9411243–11411242) and randomly chose 20 kb segments (approximately 200 SNVs) from the 2 Mb regions for each replicate. The minor allele frequencies (MAF) distributed highly skewed towards rare variants (Fig. S2). The phenotype information was simulated based on all causal genetic markers. Among 1092 individuals available from the 1000 genome project, we randomly chose 750 individuals to build the risk prediction models and used the remaining 342 subjects to evaluate the performance of the models.

#### Scenario I: The Impact of Different Weights on Methods’ Performance

In this set of simulations, we simulated two disease-associated genes (i.e. we selected 2 non-overlapped 20 kb segments from the genome), on which 33% of SNVs were causal. We further simulated three genes, none of which carried any causal SNVs. The phenotypes were simulated based on causal variants under additive model, 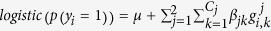, where 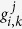 codes the number of the minor alleles for the *k*^th^ causal SNVs on the *j*^th^ gene of the *i*^th^ subject. *C*_*j*_ is the number of causal SNVs on the *j*^th^ gene, and *β*_*jk*_ is the effect size for the *k*^th^ causal SNVs on the *j*^th^ gene, 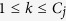.

We simulated four disease scenarios by varying the effect sizes (i.e. *β*_*jk*_). Specifically, in the first disease model (S1), the effect sizes of causal SNVs were all equal (i.e. *β*_*jk*_ = *β*_*j*_). In the second disease model (S2), the effect sizes of causal SNVs were proportional to beta weights (i.e. 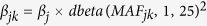, where MAF_*jk*_ is the MAF for SNV *k* on *j*^th^ gene). In the third disease model (S3), the effect sizes were proportional to the weighted sum statistics type of weights (i.e. 
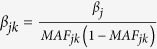
). In the last disease model (S4), the effect sizes of causal SNVs were proportional to the weights in a log functional form (i.e. 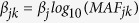).

Given four disease models with different emphasis on common or rare variants, it was apparent that the accuracy of the risk prediction models could be affected by the pre-specified weight, which represented the relative contribution of the causal SNVs to the risk of disease. In the analysis, we considered four types of weights (Table 1), corresponding to the weights used in four disease models. We evaluated the performance of FRF with the incorporated weight selection algorithm and those without weight selection (i.e. using a pre-specified weight). We further compared its performance to those of SVM and MSVM.

#### Scenario II: The Impact of Causal/Noise SNV Ratios on Methods’ Performance

In this set of simulations, we evaluated the performance of the three methods by varying the percentage of causal variants in each gene. Same as Scenario I, we considered four disease models (i.e. S1–S4) with two causal genes and three non-causal genes. For each disease model, we started with the case where all SNVs in two disease-associated genes were causal. We then gradually increased the proportion of non-causal variants on the two genes by varying causal/non-causal ratio on the two segments from1/1, 1/2, 1/4 to1/6. While applying the proposed method, we considered four weights (Table 1) for each gene. The *AUC*s of our method were then compared to those of SVM and MSVM.

#### Scenario III: The Impact of Number of Non-causal Genes on Methods’ Performance

In this set of simulations, we evaluated the performance of the three methods by gradually increasing the number of non-causal genes. Similar to Scenario I, we randomly simulated two causal genes, on which half of variants were causal. The phenotypic values were simulated according to the four disease models (i.e. S1–S4) described above. The number of non-causal genes was then increased from 3, 5, 7 to 9. Similar to the above scenarios, we considered four weights (Table 1) in the proposed method, and compared *AUC*s of our method to those of SVM and MSVM.

### Simulation results

#### Scenario I: The Impact of Different Weights on Methods’ Performance

**Figure 1:**
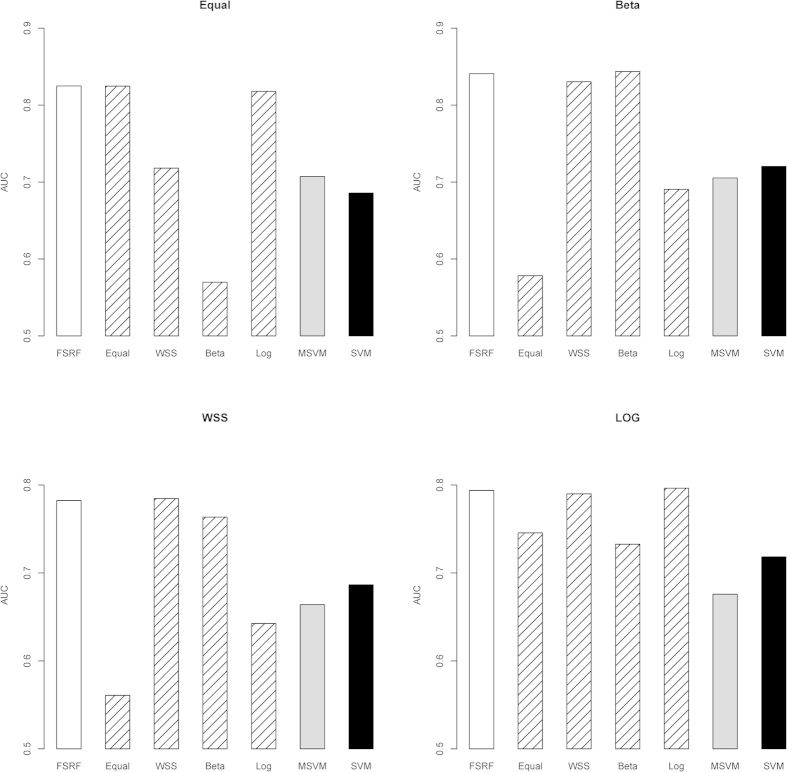
The impact of different weights on methods’ performance under various disease models.

The simulation results are illustrated in Fig. 1. Given different underlying disease models, the accuracy could be affected by the pre-specified weight. As expected, the weights that represented the underlying contribution of the variants performed the best. Ideally, the proposed method should adopt the weight that reflected the true effects of causal variants. However, in practice the relative contribution of common and rare variants were not known in advance. It is therefore necessary to allow for the flexibility of different weights in the method, which makes it robust against different underlying disease models. As shown in Fig. 1, although the FRF method did not always attain the highest accuracy for all scenarios, its performance was close to that of the best model. On average, about 91% of the times the FRF method could select the right weight. In practice, unless we have decent knowledge of the disease model, a good strategy is to consider multiple weights and let the data to determine the best weight. To further evaluate the performance of FRF method, we calculated the probability of correctly identifying non-causal genes. On average, there was 94.5% chance that our method could at least tease out one non-causal gene. Because the FRF method was capable of excluding non-causal gene, it reduced the effect of noise variants and hence increased the accuracy and robustness of the prediction model. The FRF method outperformed both SVM and MSVM as shown in Fig. 1. Regarding the computational time, on average FRF took 8 mins to analyze each replicate.

#### Scenario II: The Impact of Causal/Noise SNV Ratios on Methods’ Performance

**Figure 2:**
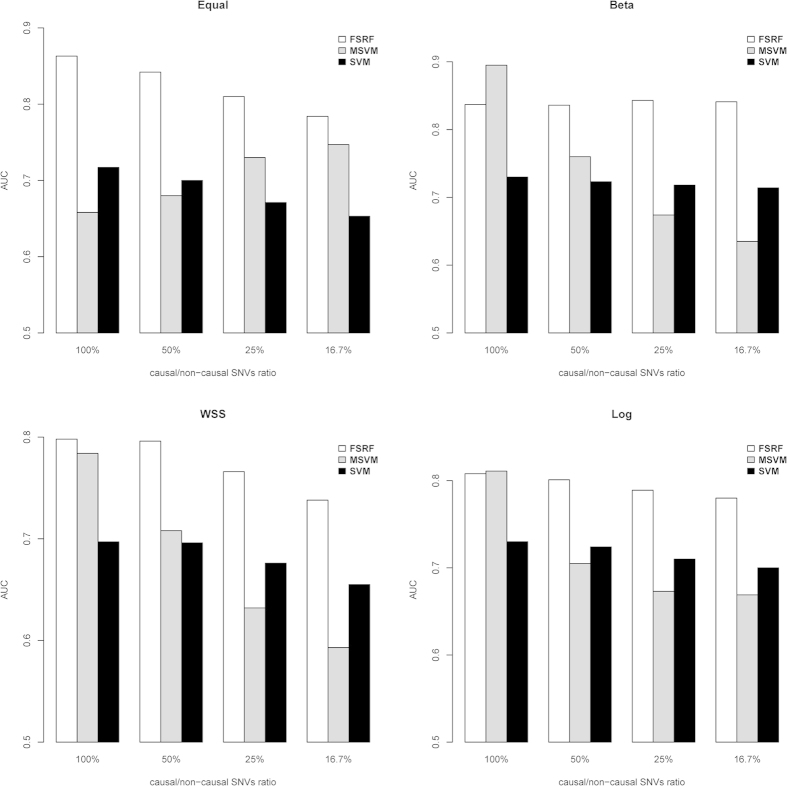
The impact of causal/non-causal SNVs ratio on methods’ performance under various disease models.

The simulation results are illustrated in Fig. 2. For disease models S2–S4, the *AUC*s of all three methods decreased as the number of noise SNVs increased. With regard to the prediction accuracy, our method performed consistently better than MSVM under disease models S2–S4, except for the case when all the markers in the genes were causal. Although the prediction accuracy for both MSVM and FRF decreased as the number of noise SNVs increased, the FRF method was less sensitive to the noise SNVs. On average, the *AUC*s dropped from 0.814 to 0.786 for FRF as the noise/causal ratio increased, while the *AUC*s dropped from 0.830 to 0.633 for MSVM (Table 2). Although most of the non-causal common variants could be excluded using the pre-selection scheme employed by the MSVM, the substantial amount of noise in rare variants may greatly influence the performance of the CMC method, leading to reduction in prediction accuracy. This especially holds when rare variants play an important role in the disease prediction. For S1 model in which all the variants had similar effects, our method outperformed the MSVM. While the performance of our method decreased with the increase in the number of noise loci, the performance of MSVM increased in the S1 model. This could be explained by the fact that when the disease was caused by common variants, the MSVM method benefited substantially by applying the pre-selection procedure on the common variants. When all SNVs were causal, the pre-selection scheme in MSVM could mistakenly exclude a small number of causal common SNVs with small to moderate effect sizes, resulting in the reduction of prediction accuracy. On the other hand, when the number of common noise SNVs was substantial, the pre-selection scheme helped to reduce the noise SNVs, which led to an improvement in the prediction accuracy. This was especially the case under disease model S1 where rare variants played a less important role in the disease risk and the disease risk was essentially determined by common variants.

Compared to the original SVM method, although the *AUC*s of FRF decreased with the increasing number of noise loci, FRF showed consistently higher accuracy than SVM under all simulated scenarios. This can be mainly explained by the fact that our method could exclude non-causal genes, which efficiently reduced noise loci.

Similar to simulation one, we further calculated the probability of correctly identifying noise genes. Although the *AUC*s decreased with the increase of noise to signal ratio, the probability of excluding at least one non-causal gene remained stable (Table 2) with an averaged value of 94.4%. Regarding the computational time, on average FRF took 9 mins to analyze each replicate.

#### Scenario III: The Impact of Number of Non-causal Genes on Methods’ Performance

**Figure 3:**
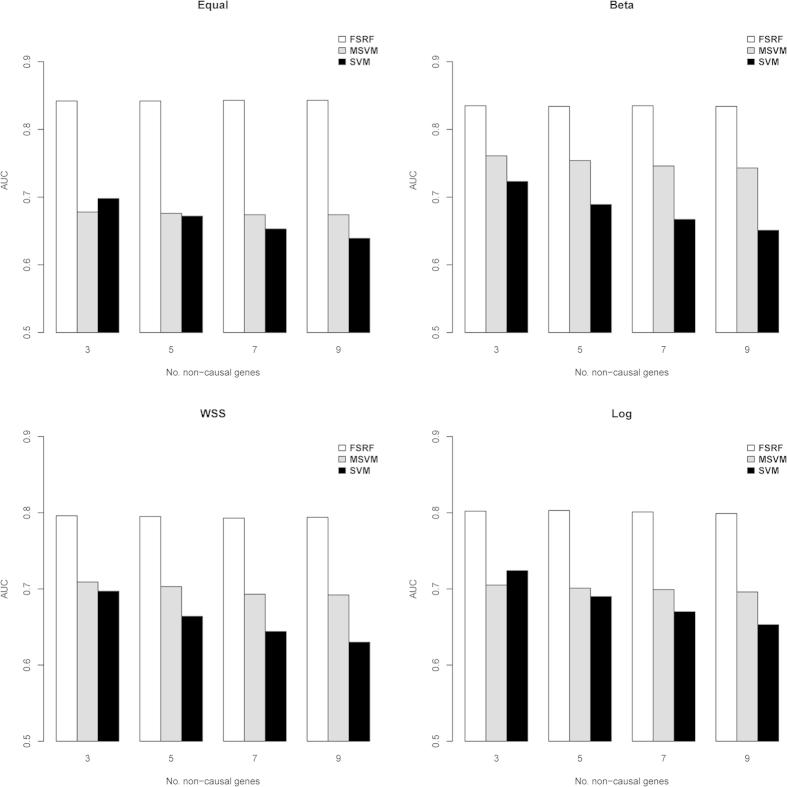
The impact of the number of non-causal genes on methods’ performance under various disease models.

The simulation results were illustrated in Fig. 3. While the *AUC*s of SVM decreased with the increasing number of non-causal genes (i.e. those with no causal SNVs), the *AUC*s of MSVM and FRF remained stable, indicating that both FRM and MSVM were robust against the number of noise genes included in the model. Although the selection procedures adopted by the FRF and MSVM methods differ in details, both of them aimed at reducing the effects of noise genes. It is worth noting that while the increase in the proportion of noise SNVs influenced the performances of both FRF and MSVM at a various degree (Scenario II), the increase in the number of noise genes rarely affected the prediction accuracy. This is because the FRF method focused on the gene-level analysis and the noise genes are likely to be excluded through the selection procedure. For MSVM, the majority of the non-causal common SNVs could be eliminated from the pre-selection procedure. Since rare variants are collapsed based on genes, the noise rare variants locate on noise gene would have little effect on risk prediction. With 50% of SNVs being causal for disease-associated genes, the FRF method outperformed the MSVM method (Scenario II). The trend preserved as the number of noise genes increased (Fig. 3). Regarding the SVM method, as expected, the prediction accuracy decreased with the increased number of noise genes. Except for a few cases in which the number of non-causal genes was small, SVM performed the worst among the three methods. Regarding the computational time, on average FRF took 8 mins, 13 mins, 22 mins and 32 mins to analyze each replicate when the number of noise genes are 3, 5, 7, and 9, respectively.

### Application to the sequence data from Dallas Heart Study (DHS)

We applied the FRF, MSVM and SVM methods to a sequencing dataset from the Dallas Heart Study^21^. The dataset comprised of four candidate genes, *ANGPTL3*, *ANGPTL*4, *ANGPTL*5 and *ANGPTL*6, all of which belonged to the *ANGPTL* family^22^. We were interested in studying the role of these four genes in predicting the high-density lipoprotein (HDL). We first re-assessed the quality of the data. We eliminated seven individuals without HDL measured, and also excluded genetic variants and individuals with a high missing rate. We further excluded variants with MAF equal to zero. After the quality control, 283 variants (72, 76, 72, and 63 variants were from *ANGPTL3*, *ANGPTL*4, *ANGPTL*5 and *ANGPTL*6, respectively) and 2591 individuals were included in the final analysis. The distributions of MAFs for the four genes are plotted in Fig. S3, which were highly skewed to the rare variants with the majority of the variants (87%) having MAF < 1%. We categorized the HDL into two levels, with HDL <= 40 being in the low HDL level (N = 704) and HDL > 40 being in the median to high HDL level (N = 1887)^23^.

**Figure 4:**
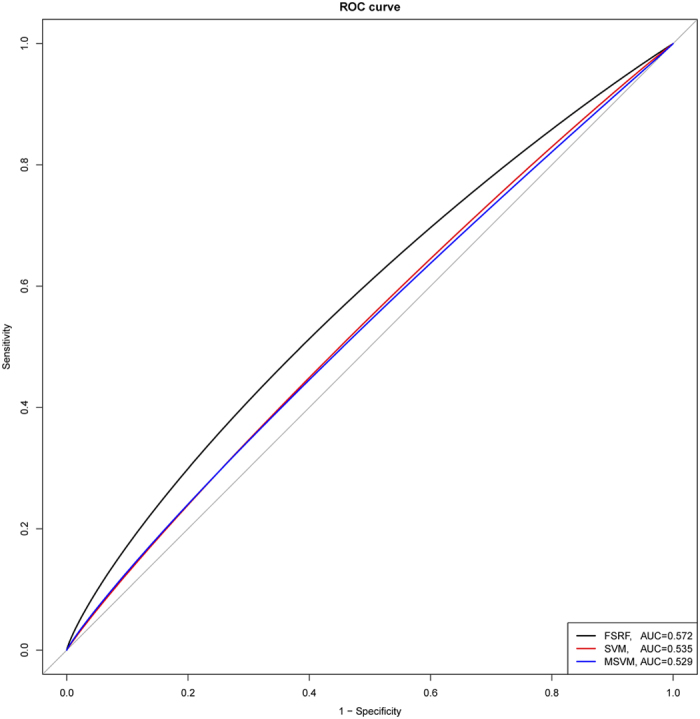
ROC curves of three prediction models formed by FRF, SVM and MSVM using the DHS sequencing data.

To evaluate the three methods, we randomly selected 75% of samples to train the model and used the remaining samples to evaluate the model. The FRF method selected *ANGPTL4*, *ANGPTL5* and *ANGPTL6* into the final model, and *AUC* of the proposed method was 0.572. We further applied the MSVM method and the SVM method to the data, and the *AUC*s are 0.529 and 0.535, respectively. The receiver operating characteristic (ROC) curves generated by the three methods are plotted in Fig. 4. From this figure, we can easily visualize that the risk prediction model built by FRF attained higher accuracy than the models from the rest two methods. To avoid the chance finding due to randomly splitting of the data, we repeated the process 100 times. The mean *AUC*s and the standard errors are summarized in Table 3. While the three methods had a comparable standard error (around 0.02), the FRF method achieved the highest AUCs (0.570). As the prediction models built by SVM based methods were hard for interpretation, we only focused on interpreting the prediction model built by the FRF method. Among 100 repeats, *ANGPTL*4, *ANGPTL*5 and *ANGPTL*6 were selected 99, 91 and 89 times, respectively. More than 80% of times the Beta and WSS weights were selected for *ANGPTL*4, *ANGPTL*5 and *ANGPTL*6, which indicated that the rare variants in these three genes played a substantial role in the HDL prediction.

## Discussion

A random field is a generalization of a stochastic process that takes multidimensional points with specific structure. The theory in random field has been extensively studied and the methods using random field have been widely used in spatial analysis^16^,^17^,^18^. The general idea of random field methods is to predict the outcome at a given point through a weighted average outcome based on its surrounding points^24^. Despite its wide application in spatial statistics, random field has been rarely used for high dimensional genetic risk prediction research. In this paper, we proposed a FRF method within the random field framework for risk prediction using sequencing data. FRF method is built based upon the idea that the similarities in genetic profiles would lead to the similarities in the phenotypes. In FRF, each individual can be mapped into a space based on the individual’s genotypic profile, and the distance of the individual to other individuals in the space can be measured with pre-specified distance functions. Based on this geometric structure, we can predict the individual’s phenotype based on a weighted average of phenotypes from nearby individuals, where the weight is determined by the distance of two individuals.

Rare variants play an important role in the underlying mechanism of human disease, and hold great promise to further improve the accuracy of the prediction model^6^,^7^,^8^,^9^,^25^. However, due to the large number, low frequencies, and unknown effects of rare variants on diseases, the conventional method can hardly capture the effects of rare variants, making it hard to incorporate rare variants into risk prediction models. In the analysis of sequencing data, a variety of weight/kernel functions have been proposed to adjust for various effects of rare variants, but none of them performed uniformly better than the others^26^. Both our simulations and previous studies have shown that the performance of a weight function was determined by the underlying disease model^26^. The ideal choice of weights should be those that reflect the underlying disease model, however, in practice it is usually unknown. Instead of pre-specifying a specific weight function, the FRF method allows for various weight functions, and then uses a forward selection algorithm to select a weight function best measuring the genetic similarities of individuals. Through simulations, we have demonstrated that the FRF method performed close to the model in which the underlying weight was specified. This suggests that the FRF method can adaptively choose the optimal weight for the similarity measure, and make the formed risk prediction model robust against different underlying disease models. Although the method was illustrated with weight selection, it could be easily extended to similarity function selection. Instead of pre-specifying a similarity function for a gene, we could evaluate various functions and let the algorithm to adaptively select the best function.

Different genes could have different effects on the disease mechanism. Some genes serve as protective roles to prevent the disease onset, while the others increase disease risk and accelerate the disease progression^27^,^28^. Genes may also have no contribution to disease, especially for large studies with many measured genes. In such a case, including these non-disease-associated genes may add a substantial amount of noise and reduce the accuracy of the prediction model. To address this issue, the proposed method allows for multiple genes with different effect sizes to be simultaneously considered, and jointly estimates the effect of each gene by controlling the effects of the others. The FRF method also adopts a computationally efficient forward selection algorithm, which makes it possible to reduce the effects of noise genes and be applied to the genome-wide data. Through simulations, we have demonstrated that ruling out the noise genes improved the performance of the risk prediction model, and made the method less sensitive to the noise genes. Compared with the SVM based methods, the findings from the FRF method can be easily interpreted, as it selects predictors and predicts the phenotypes at the gene-level. The genes that are selected by the FRF method could be treated as the functional units that predict phenotype, which facilitates future studies to confirm and further explore the properties (e.g., predictiveness) of the risk prediction model.

Although the proposed method allows for both gene and weight selection, it cannot remove noise markers located on disease-associated genes. Methods that are capable of selecting variants within a gene can be further developed to improve the accuracy of a prediction model. Nevertheless, as shown from the simulations FRF was generally robust to the presence of noise SNVs as compared with the other SVM based methods.

In the empirical study of DHS, we applied the FRF method to four genes to predict HDL. We randomly chose 75% of the samples to serve as the training set, and used the remaining samples to assess the performance of the formed prediction model. The FRF selected three genes into the model, and the *AUC* on the testing dataset was 0.572. It has been shown that genes in the *ANGPTL* family were regulators of lipoprotein metabolism in humans, and the rare loss-of-function mutations in *ANGPTL* family members may contribute to HDL^21^,^22^,^29^. However, so far there is limited knowledge on how rare variants contribute to HDL prediction. Additional studies are needed to validate this preliminary finding and further evaluate the role of rare variants in HDL prediction.

## Additional Information

**How to cite this article**: Wen, Y. *et al.* Risk Prediction Modeling of Sequencing Data Using a Forward Random Field Method. *Sci. Rep.* **6**, 21120; doi: 10.1038/srep21120 (2016).

## Supplementary Material

Supplementary Information

## Figures and Tables

**Table 1:** Four weight functions considered in our study.

Un-weighted (UW)	Beta distribution type of weights (BETA)	Weighted sum statistics type of weights (WSS)	Logarithm of MAFs as weights (LOG)
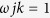	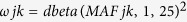	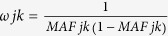	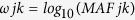

**Table 2:** The impact of varied causal/non-causal SNVs ratio on the performance of three methods under different disease models.

Disease Model	% of causal markers	FRF	MSVM	SVM	Probability*
Equal	100	0.863	0.658	0.717	0.941
	50	0.842	0.680	0.700	0.940
	25	0.810	0.730	0.671	0.951
	16.7	0.784	0.747	0.653	0.944
Beta	100	0.837	0.895	0.730	0.947
	50	0.836	0.760	0.723	0.950
	25	0.843	0.674	0.718	0.945
	16.7	0.841	0.635	0.714	0.936
WSS	100	0.798	0.784	0.697	0.951
	50	0.796	0.708	0.696	0.954
	25	0.766	0.632	0.676	0.947
	16.7	0.738	0.593	0.655	0.945
LOG	100	0.808	0.811	0.730	0.945
	50	0.801	0.705	0.724	0.944
	25	0.789	0.673	0.710	0.950
	16.7	0.780	0.669	0.700	0.938

^*^The probability of excluding at least one non-disease-related gene.

**Table 3:** The *AUC* values of the three prediction models formed by FRF, SVM and MSVM using the DHS sequencing data.

	FRF	SVM	MSVM
Mean	0.570	0.528	0.529
Standard Error	0.022	0.021	0.024
