# Supplementary material for: Risk Prediction Modeling of Sequencing Data Using a Forward Random Field Method
Source: Sci Rep. 2016 Feb 19;6:21120. doi: 10.1038/srep21120 (PMC4759688; doi:10.1038/srep21120)
Supplement: Supplementary Information [file srep21120-s1.pdf]

Risk Prediction Modeling of Sequencing Data Using a Forward Random Field Method

Yalu Wen, Zihuai He, Ming Li and Qing Lu

Supplementary Information

1. For each gene $l = 1,2,\cdots,L$

   For each kernel/weight $w = 1,2,\cdots,W$

   Fit model for each gene and each kernel/weight using training dataset.

   Calculate risk score for individuals in training dataset and calculate the AUC.

   End loop for l, w.

   Choose the l and w that maximize the AUC.

2. For each gene $l = 1,2,\cdots,L$

   For each kernel/weight $w = 1,2,\cdots,W$

   Together with the genes selected from previous steps, add genes and kernels/weights one at a time into the model.

   Fit model using training dataset.

   Calculate risk score for individuals in training dataset and calculate the AUC.

   End loop for l, w.

   Choose the l and w that maximize the AUC .

3. Repeat step 2 until the optimum number of genes or a pre-specified number of genes (based on prior biological knowledge) are selected.

Figure S1. The flowchart of the forward selection algorithm

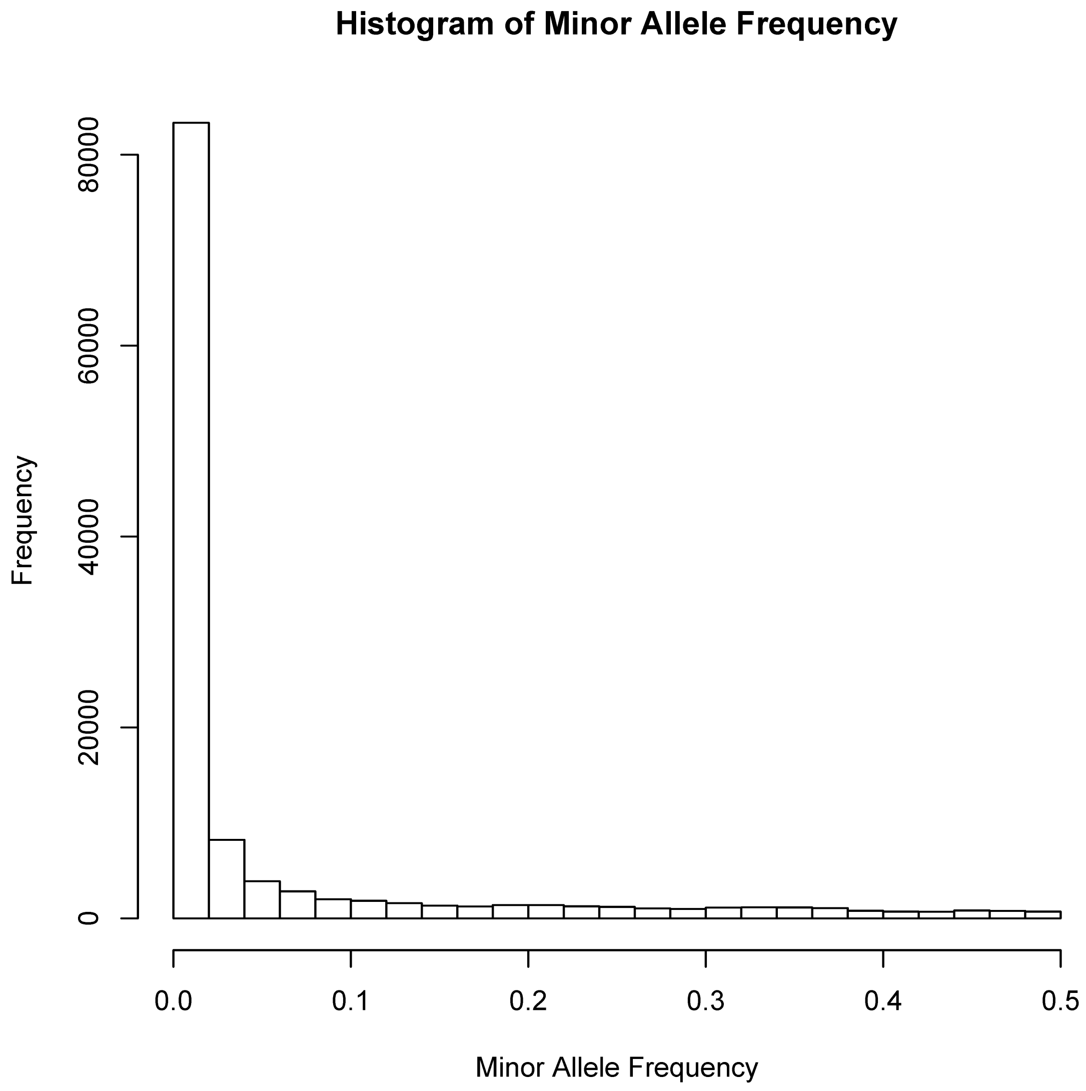

Figure S2. Minor allele frequency distribution of variants used in the simulations

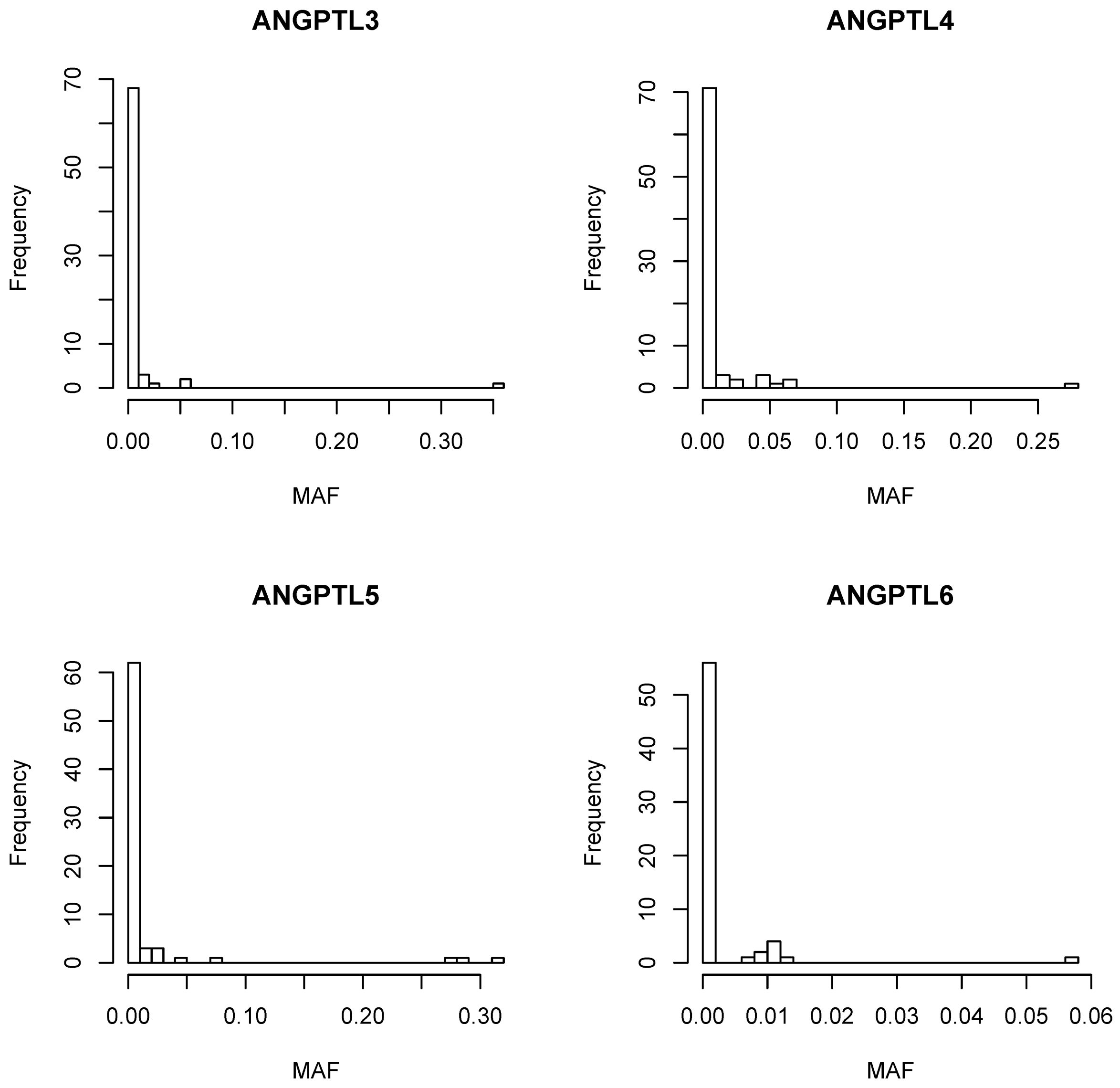

Figure S3. Minor allele frequency distribution of variants in four genes from the Dallas Heart Study
